# Pastoral Letter by Bishop Coxe

**Published:** 1869-02

**Authors:** A. Cleveland Coxe

**Affiliations:** Bishop of Western New York, and in charge of the Diocese of Central New York


### Pastoral Letter by Bishop Coxe.

We desire to extend to the profession, this much of Bishop Coxe's Pastoral Letter to the clergy and laity of Western and Central New York. "If there be a special damnation for the clergy and laity who shed innocent blood," what must be the portion of physicians who aid and countenance infanticide, with a full knowledge of the magnitude and extent of the crime?

Dearly beloved, save yourselves and this untoward generation, from such unnatural, unspeakable crime and villainy.

I have heretofore warned my flock against the blood-guiltiness of ante-natal infanticide. If any doubts existed heretofore as to the propriety of my warnings on the subject, they must now disappear before the fact that the world itself is beginning to be horrified by the practical results of the sacrifices to Moloch which defile our land. [I ask attention to an article on "Population," which appears in a periodical called *Harpers' Magazine*, for February, 1869.] Again I warn you that they who do such things cannot inherit eternal life. If there be a special damnation for those who "shed innocent blood," what must be the portion of those who have no mercy upon their own flesh?

Dearly beloved, "save yourselves from this untoward generation."

Your affectionate Bishop,

A. CLEVELAND COXE,

Bishop of Western New York, and in charge of the Diocese of Central New York.

